# Use of RNA-Protein Complexes for Genome Editing in Non-*albicans Candida* Species

**DOI:** 10.1128/mSphere.00218-17

**Published:** 2017-06-21

**Authors:** Nora Grahl, Elora G. Demers, Alex W. Crocker, Deborah A. Hogan

**Affiliations:** Department of Microbiology and Immunology, Geisel School of Medicine at Dartmouth, Hanover, New Hampshire, USA; Carnegie Mellon University

**Keywords:** CRISPR, *Candida*, *auris*, genome editing, *glabrata*, *lusitaniae*, molecular methods

## Abstract

Clustered regularly interspaced short palindromic repeat** **(CRISPR)-Cas9 genome modification systems have greatly facilitated the genetic analysis of fungal pathogens. In CRISPR-Cas9 genome editing methods designed for use in *Candida albicans*, DNAs that encode the necessary components are expressed in the target cells. Unfortunately, expression constructs that work efficiently in *C. albicans* are not necessarily expressed well in other pathogenic species within the genus *Candida* or the related genus *Clavispora*. To circumvent the need for species-specific expression constructs, we implemented an expression-free CRISPR genome editing system and demonstrated its successful use in three different non-*albicans Candida* species: *Candida* (*Clavispora*)* lusitaniae*, *Candida glabrata*, and *Candida auris*. In CRISPR-Cas9-mediated genome editing methods, a targeted double-stranded DNA break can be repaired by homologous recombination to a template designed by the investigator. In this protocol, the DNA cleavage is induced upon transformation of purified Cas9 protein in complex with gene-specific and scaffold RNAs, referred to as RNA-protein complexes (RNPs). In all three species, the use of RNPs increased both the number of transformants and the percentage of transformants in which the target gene was successfully replaced with a selectable marker. We constructed mutants defective in known or putative catalase genes in *C. lusitaniae*, *C. glabrata*, and *C. auris* and demonstrated that, in all three species, mutants were more susceptible to hydrogen peroxide than the parental strain. This method, which circumvents the need for expression of CRISPR-Cas9 components, may be broadly useful in the study of diverse *Candida* species and emergent pathogens for which there are limited genetic tools.

**IMPORTANCE** Existing CRISPR-Cas9 genome modification systems for use in *Candida albicans*, which rely on constructs to endogenously express the Cas9 protein and guide RNA, do not work efficiently in other *Candida* species due to inefficient promoter activity. Here, we present an expression-free method that uses RNA-protein complexes and demonstrate its use in three *Candida* species known for their drug resistance profiles. We propose that this system will aid the genetic analysis of fungi that lack established genetic systems.

## Abstract (precis)

Existing CRISPR-Cas9 genome modification systems for use in *Candida albicans*, which rely on constructs to endogenously express the Cas9 protein and guide RNA, do not work efficiently in other *Candida* species due to inefficient promoter activity. Here, we present an expression-free method that uses RNA-protein complexes and demonstrate its use in three *Candida* species known for their drug resistance profiles. We propose that this system will aid the genetic analysis of fungi that lack established genetic systems.

## INTRODUCTION

In the past few years, clustered regularly interspaced short palindromic repeat (CRISPR) genome editing, a process first discovered through the study of bacterium-bacteriophage interactions (1), has emerged as a major strategy for genome editing in eukaryotes (2, 3). CRISPR-Cas9 genome modification methods rely on a nuclease, Cas9, to make a precise double-stranded DNA break which can then be repaired by nonhomologous end joining, causing a potential insertion or deletion, or homologous recombination using a construct with homology both up- and downstream of the cut site. Repair constructs contain selectable markers and can be engineered to introduce genome changes such as gene replacements, protein tags, or other directed mutations (4–6). Cas9 interacts with the guide RNA which directs Cas9 to the cut site by hybridizing to the gene of interest with a 20-bp target-specific guide or protospacer sequence. Cas9-mediated cutting occurs only if the protospacer sequence is directly followed by a PAM (protospacer adjacent motif) site, an essential targeting component of the CRISPR-Cas9 system, which distinguishes bacterial self from nonself DNA in phage resistance (1–3).

The study of pathogenic fungi, including *Candida albicans*, has been enhanced by the introduction of strategies for genome modification via CRISPR-Cas9 systems. CRISPR-Cas9-mediated modification of *C. albicans* was first reported by Vyas et al. (7), using a method that employed the stable expression of the Cas9-encoding gene from a construct located on the chromosome. Later iterations of CRISPR-Cas9 modification of *C. albicans* demonstrated the use of transient gene expression systems wherein DNAs encoding Cas9 and guide RNAs could be cotransformed with the repair construct (8). In these highly effective published systems, designed for use in *C. albicans*, the Cas9 protein is derived from DNA constructs in which the *CAS9* gene is under the control of a promoter known to be well expressed in *C. albicans*. Recent studies performed by Norton et al. (see reference 9) have shown that constructs designed for use in *C. albicans* do not lead to efficient transformation in *Candida* (*Clavispora*)* lusitaniae* due to differences in promoter requirements. A CRISPR-Cas9 system has also been recently established for *Candida glabrata*, but again, this system relies on the use of species-specific promoters from either *Saccharomyces cerevisiae* or *C. glabrata* (10). A strategy that does not require the availability of established promoters and terminators for each species could enhance research in diverse fungal pathogens.

Genome editing research in human cells has demonstrated the successful use of a CRISPR-Cas9 method utilizing purified Cas9 protein and CRISPR RNAs, rather than *CAS9*- and guide RNA-expressing constructs (11). Expression-free CRISPR-Cas9 genome editing has also recently been demonstrated in plants (12), algae (13), and the filamentous fungus *Penicillium chrysogenum* after protoplast formation (14). This method has not yet been applied to pathogenic yeast.

Here, we present genomic modification of *C. lusitaniae*, *Candida auris*, and *C. glabrata*, three species that are notorious for innate or acquired antifungal resistance (15–20), using CRISPR RNA-Cas9 protein complexes (RNPs) along with a repair construct that contains the desired genome modification. The inclusion of RNPs increased both the number of transformants and the percentage of transformants with the desired mutation in all three *Candida* species. Using RNP-mediated genome editing, we constructed mutants lacking either a known or a putative catalase gene(s), and in all three species, the deletion of the selected catalase gene led to increased sensitivity to hydrogen peroxide as expected. We expect that this method will be useful for the genetic analysis of diverse fungal species that lack established gene expression systems.

## RESULTS

### Strategy for RNP-mediated deletion of known or putative catalase genes in diverse *Candida* spp.

The lack of an established gene expression system for CRISPR-Cas9-mediated genome editing slows research on emerging pathogens. In this study, we sought to determine if RNPs, which contain the protein and RNA components of the CRISPR-Cas9 system, could be used to make genetic alterations in three diverse fungal pathogens without the need for defined promoters for heterologous gene expression. To do this, we designed a strategy to knock out proposed or validated catalase-encoding genes in *C. lusitaniae*, *C. glabrata*, and *C. auris*. The gene encoding catalase was targeted because the null mutant phenotype is easily assayed, thus facilitating these proof-of-principle experiments, and because the catalase mutants may be useful in future studies on the role of reactive oxygen species (ROS) in killing by antifungal compounds or through interactions with the host immune system (21, 22).

**FIG 1:**
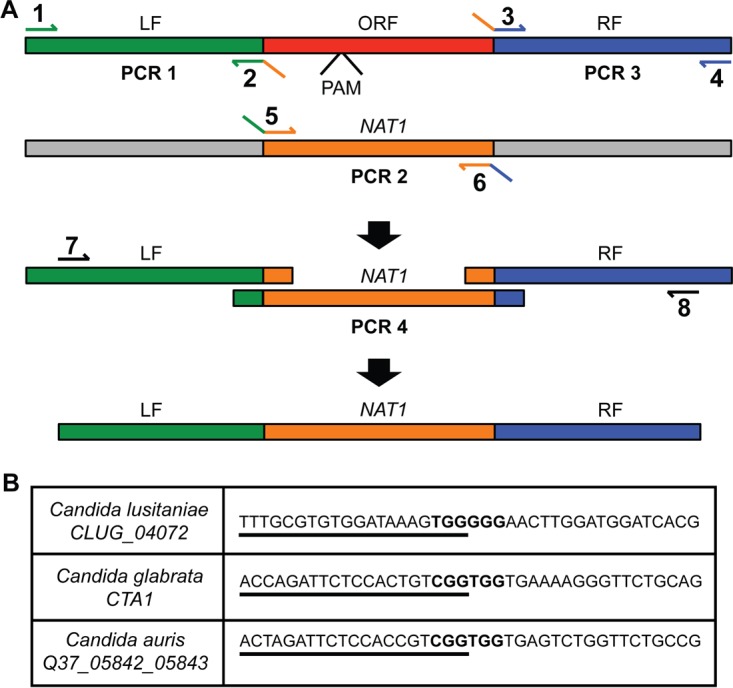
Scheme for creating the gene deletion constructs and gene-specific crRNAs. (A) The components needed to create the gene deletion cassettes were generated in three PCRs. The locus or ORF to be deleted is shown in red, and the nourseothricin resistance gene (*NAT1*) is shown in orange. Regions of 500 to 1,000 bp flanking the target ORF (left flank [LF] and right flank [RF]) were amplified using the primers shown in PCRs 1 and 3. The *NAT1* cassette was amplified in PCR 2 with primers 5 and 6. Primers 2 and 3 contained reverse-complemented sequences to primers 5 and 6 that were used to fuse the *NAT* cassette to the LF and RF amplicons in the PCR 4 stitching reaction with nested primers 7 and 8. The resulting gene deletion construct was used for transformation. (B) The gene-specific part of the crRNA is a 20-bp sequence (underlined) that ends with a CRISPR-Cas9 PAM site and is directly adjacent to an additional PAM site (PAM sites shown in bold).

Genes in *C. lusitaniae*, *C. glabrata*, and *C. auris* with the highest level of homology to the gene encoding *C. albicans* catalase, *CAT1* (C1_06810W_A), were identified by BLAST (23). *C. lusitaniae CLUG_04072*, an uncharacterized gene, and *C. glabrata CTA1* (24), which has been previously characterized as catalase (25), were found. In *C. auris*, the gene with the highest homology to *C. albicans CAT1* (*CaCAT1*) was hypothetical gene QG37_05842, but subsequent protein sequence alignment of this open reading frame (ORF) to the known and putative catalases of *C. albicans*, *C. glabrata*, and *C. lusitaniae*, performed using Clustal Omega (26), suggested that this initial annotation did not encompass the full-length protein. This analysis strongly suggested that the ORFs annotated as QG37_05842 and QG37_05843 are actually one gene that encodes a catalase (see Fig. S1 in the supplemental material). To create the constructs necessary to delete the catalase-encoding genes, PCR was used to fuse a selectable nourseothricin resistance marker, *NAT1*, with DNA sequence from upstream and downstream of the indicated catalase genes. The *NAT1* gene was codon optimized for use in *Candida* spp. (27). The amount of homologous sequence used varied slightly between constructs, ranging from 500 to 1,000 bp. The final constructs contained 896 and 984 bp for *C. lusitaniae*, 977 and 968 bp for *C. auris*, and 818 and 531 bp for *C. glabrata* for the left and right flanks, respectively. The methods for generating these gene deletion constructs are outlined in Fig. 1A.

10.1128/mSphere.00218-17.1 FIG S1  Alignment to compare the catalase protein sequences of *C. lusitaniae*, *C. glabrata*, and *C. auris* to *Candida albicans* Cat1. Protein sequences were retrieved from the *Candida* genome database or NCBI, and sequences were aligned using the Clustal Omega multiple sequence alignment tool. The two *C. auris* ORF annotations that made up the *CAT1* gene are indicated in red (*QG37_0543*) or blue (*QG37_05842*). Download FIG S1, PDF file, 0.4 MB. Copyright © 2017 Grahl et al. 2017 Grahl et al. This content is distributed under the terms of the Creative Commons Attribution 4.0 International license.

In the CRISPR genome modification method tested here, the CRISPR machinery that is cotransformed with the gene deletion construct cleaves the chromosome at a site between the regions where homologous recombination is desired. The CRISPR machinery includes purified Cas9 protein and two RNAs: the CRISPR guide RNA (crRNA), which contains 20 bp homologous to the target gene fused to the scaffold sequence, and a universal transactivating CRISPR RNA (tracrRNA), which forms an RNA duplex with the gene-specific crRNA and subsequently complexes with the Cas9 nuclease. To ensure that the protein and RNA components are properly assembled and transformed together, the crRNA and tracrRNA are coincubated and then added to purified Cas9 protein, allowing formation of the RNA-protein complex prior to electroporation. Typically, crRNAs are designed with homology to 20 bp adjacent to a PAM site (NGG). However, to maximize knockout efficiency, we designed the crRNA used in this study to target adjacent PAM sites (NGGNGG) as this dramatically increases CRISPR efficiency in other species (28). Thus, the gene-specific crRNAs used below recognize a 20-bp sequence that ends with a CRISPR-Cas9 PAM site (7) and is directly adjacent to an additional PAM site (Fig. 1B). NCBI BLAST was used to verify that the chosen gene-specific 20 bp had no off-site targets in the genome of the respective fungal pathogen.

### RNPs increase the efficiency of *C. lusitaniae CLUG_04072* gene replacement.

**FIG 2:**
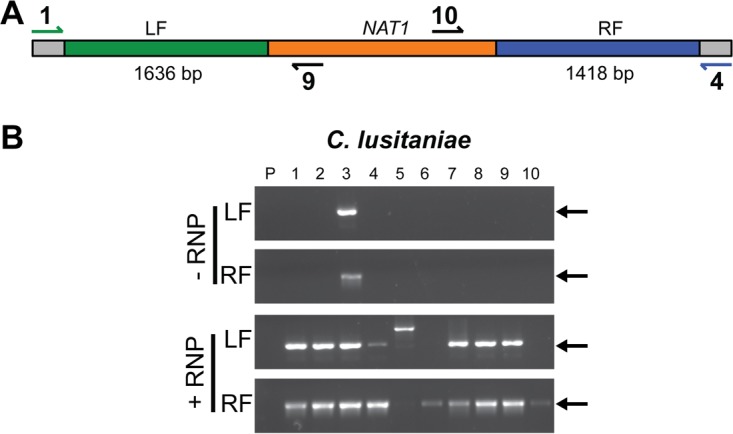
PCR genotype analysis to determine RNP-mediated knockout efficiency in *C. lusitaniae*. (A) Schematic indicating the locations of primers used to detect transformants with the *C. lusitaniae CLUG_04072Δ*::*NAT1* genotype. Amplification of the left and right flanking regions (LF and RF, respectively) was performed using sets of primers that included one annealing within the *NAT1* locus and another annealing to the genome immediately outside the deletion construct flank (depicted in blue or green). The *NAT1* gene is shown in orange. The predicted sizes for the LF and RF amplicons are shown. (B) Amplicons from reactions using primers 1 and 9 (LF) and primers 4 and 10 (RF) are shown. Genomic DNA isolated from the parental strain (P) and 10 randomly selected NAT^r^ colonies (1 to 10) from transformation reactions that included either the gene deletion construct and CRISPR RNPs (+RNPs) or the gene deletion construct alone (−RNP) was used as the template. Transformants for which there is a band present in both the LF and RF reactions were considered positive transformants with the *NAT1* gene properly integrated at the *CLUG_04072* locus. Black arrows indicate the location of the correct band size for each amplicon.

Recent studies have indicated that *C. lusitaniae* is refractory to the CRISPR method developed for *C. albicans* (9); thus, we sought to test whether RNPs could increase accurate transformation efficiency. The haploid clinical *C. lusitaniae* strain A04 was transformed with the *CLUG_04072* knockout construct in the presence and absence of *CLUG_04072*-targeted RNPs (prepared as described in Materials and Methods). As shown in Table 1, inclusion of *CLUG_04072*-targeted RNPs in the transformation significantly increased the number of nourseothricin-resistant (NAT^r^) *C. lusitaniae* transformants, resulting in approximately 10 times more transformants than transformation with the deletion construct alone. Ten NAT^r^ colonies were randomly chosen for genotype assessment from each transformation reaction using a PCR-based strategy outlined in Fig. 2A. Using the presence of the correct-size band for both the left and right flanking regions of the knockout construct as an indication of correct construct integration, we determined that only 1 out of 10 transformants obtained in the absence of RNPs was correct, whereas 7 out of 10 transformants obtained with RNPs were correct (Fig. 2B). Thus, in *C. lusitaniae* the addition of gene-specific RNPs to a transformation drastically increases the number of accurate transformants obtained.

**TABLE 1:** Transformation efficiency with and without RNPs in three *Candida* species

Species	Sample no.	Gene deletion construct	RNP	No. of Nat^r^ transformants^a^	*P* value for difference between + and − RNPs^b^
*C. lusitaniae*	1	+	−	12	
*C. lusitaniae*	2	+	+	112	<0.05
*C. auris*	3	+	−	680	
*C. auris*	4	+	+	3,880	<0.05
*C. glabrata*	5	+	−	1,857	
*C. glabrata*	6	+	+	36,400	<0.01

a The number of transformants represents the average from 3 or 4 independent transformations.

b Significance determined by ratio-paired *t* test.

### RNP-mediated CRISPR-Cas9 gene editing increases the efficiency of gene replacement in *C. glabrata* and *C. auris.*

**FIG 3:**
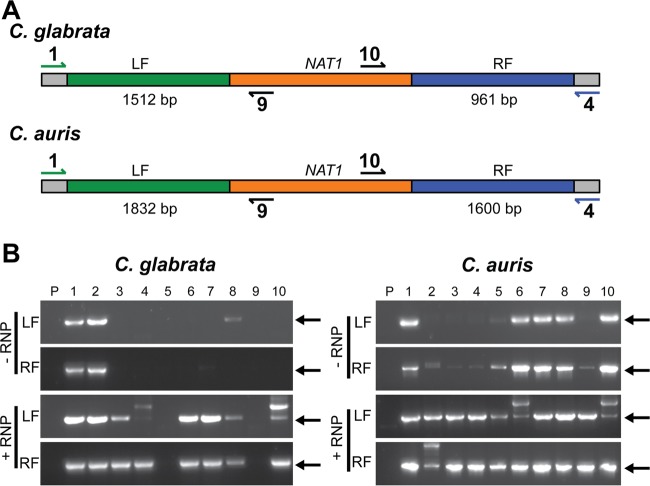
PCR genotype analysis to determine RNP-mediated knockout efficiency in *C. glabrata* and *C. auris*. (A) Schematic indicating the locations of primers used to detect *C. glabrata* and *C. auris* transformants with *cta1*Δ::*NAT1* and *QG*_*05842_05843*Δ::*NAT1* genotypes, respectively. Amplification of the left and right flanking regions (LF and RF, respectively) was performed using sets of primers that included one annealing within the *NAT1* locus and another annealing to the genome immediately outside the deletion construct flank (depicted in blue or green). The *NAT1* gene is shown in orange. The predicted sizes for the LF and RF amplicons are shown. (B) Amplicons from reactions using primers 1 and 9 (LF) and primers 4 and 10 (RF) are shown. Genomic DNA isolated from the parental strain (P) and 10 randomly selected NAT^r^ colonies (1 to 10) from transformation reactions that included either the gene deletion construct and CRISPR RNPs (+RNPs) or the gene deletion construct alone (−RNP) was used as the template. Transformants for which there is a band present in both the LF and RF reactions were considered positive transformants with the *NAT1* gene properly integrated at either the *CTA1* or *QG_05842_05843* locus in *C. glabrata* and *C. auris*, respectively. Black arrows indicate the location of the correct band size for each amplicon.

To determine if RNPs also increase the efficiency of CRISPR-Cas9-mediated gene replacement in clinical strains of *C. auris* and *C. glabrata*, we performed a similar analysis in strains of these species. As for the transformation of *C. lusitaniae*, the use of RNPs significantly increased the total number of transformants in both species (Table 1). Again, using the presence of the correct-size band for both the left and right flanking regions of the knockout construct as an indication of correct construct integration, we determined that out of 10 randomly selected NAT^r^ transformants, the addition of gene-specific RNPs increased the number of accurate transformants from two to six in *C. glabrata* and five to seven in *C. auris* (Fig. 3). Therefore, not only is the number of transformants higher but also the percentage of transformants with the marker integrated at the correct locus is increased by the addition of CRISPR-Cas9 RNPs.

### Mutants lacking known or putative catalase-encoding genes have increased hydrogen peroxide sensitivity.

**FIG 4:**
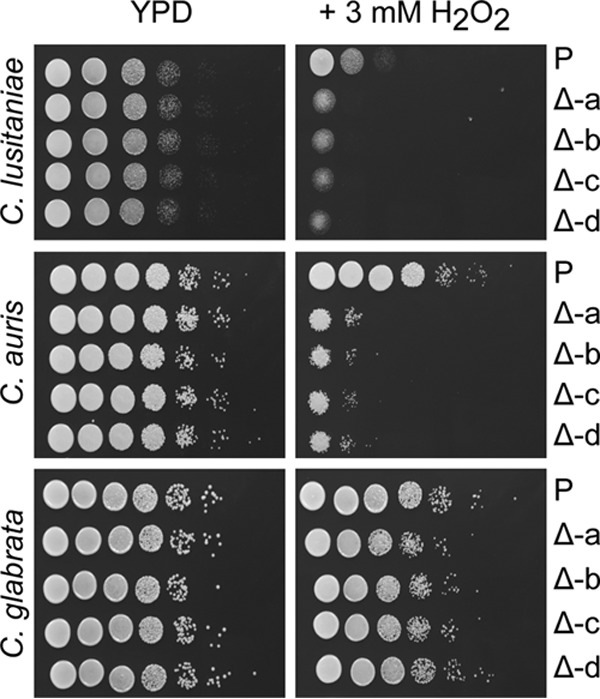
Comparison of parental strains and transformants lacking catalase genes in *C. lusitaniae*, *C. auris*, and *C. glabrata*. Comparison of serially diluted parental strain (P) and four confirmed catalase knockouts (Δ-a to Δ-d), verified by the method demonstrated in Fig. 2 and 3. Images were captured after 24 h of growth on either YPD or YPD plus 3 mM H_2_O_2_ as indicated.

We analyzed the catalase phenotypes for four confirmed mutants in each of the three *Candida* species included in these studies by plating a dilution series of each strain on agar medium with and without 3 mM hydrogen peroxide (H_2_O_2_). In all three species, deletion of the selected catalase-encoding gene did not affect growth in the absence of H_2_O_2_ but did result in decreased growth in the presence of H_2_O_2_ (Fig. 4). The most striking effect was observed in *C. auris*, in which the parental strain was highly resistant to 3 mM H_2_O_2_ and loss of QG37_05842_05843 caused a 10^4^-fold decrease in growth in all mutants tested; based on these results, we now refer to the QG37_05842_05843 loci as *CAT1*. While the parental *C. lusitaniae* isolate was comparatively more sensitive to H_2_O_2_ than *C. auris*, deletion of *CLUG_04072* still conferred increased sensitivity to H_2_O_2_. Based on the high sequence similarity to catalase sequences in other *Candida* spp. and the increased sensitivity of the *CLUG_04072*Δ strain to H_2_O_2_, we also renamed this locus *CAT1*. Consistent with previous publications, *C. glabrata*, like *C. auris*, was highly resistant to H_2_O_2_ (25), and in this strain, deletion of *CTA1* had a more modest effect on H_2_O_2_ resistance, though the effects were consistent across mutants and reproducible across experiments. The H_2_O_2_ susceptibility phenotypes of the *C. glabrata cta1Δ* strains are consistent with results reported for a *cta1Δ* mutant constructed as described in the work of Cuéllar-Cruz et al. (25).

## DISCUSSION

We propose that the methods described here will advance our ability to study pathogens of medical importance that currently lack well-developed systems for their genetic manipulation. One of the benefits of this approach is the ability to use commercially available Cas9 protein and custom-synthesized RNAs; thus, no additional lab equipment or techniques are required, and only the deletion construct needs to be synthesized by the investigator. Both design of the RNA and the deletion construct can be accomplished with minimal knowledge about an organism’s biology; the only data necessary for these steps are the gene sequence plus and minus approximately 1,000 bp. We have demonstrated that the delivery of the mixture of Cas9 protein and guide RNAs with the deletion construct enhances the number of total NAT^r^ transformants as well as the percentage of transformants with the correctly integrated marker, even in species like *C. lusitaniae*, which we, and others, have had extreme difficulties mutagenizing using non-CRISPR-Cas9-based methods. Parallel studies by Bennett and colleagues (see reference 9) highlight the inefficiency of a transient expression system developed for use in *C. albicans* when used in *C. lusitaniae* and show that efficiency increases when a species-specific promoter is used to drive the expression of the Cas9-encoding gene. Thus, the RNP-based method may be particularly impactful for species for which promoters that can drive the expression of the *CAS9* gene have not been constructed, including *C. auris*, in which multidrug-resistant strains have emerged multiple times and caused hospital-associated outbreaks (15, 17, 29, 30), and *C. glabrata*, known for its innate resistance to the most commonly used antifungals (16). In this report, we demonstrate, for the first time, that *C. auris* is naturally capable of efficient recombination. However, even in *C. auris*, the inclusion of Cas9 and guide RNAs increased the percentage of transformants with the correct gene replacement, thereby reducing the number of transformants that need to be screened. These studies have built upon the prior work by Vyas and colleagues (7), who developed the guide RNA selection strategy; Min and colleagues, who further characterized the use of CRISPR-Cas9 in *C. albicans* (8); and Shen and colleagues, who developed the optimized nourseothricin resistance marker for use in fungal species that use the CUG codon to encode serine instead of leucine (27).

To demonstrate the effectiveness of this method, we chose to delete catalase-encoding genes because although catalase-encoding genes are not essential to cellular growth under standard laboratory conditions, they are found among many diverse organisms; thus, we expected to identify putative catalase genes in *C. lusitaniae* and *C. auris*. In addition, not only is the loss of catalase activity easily assessed phenotypically, allowing us to show that the genetic evidence of gene deletion matches an expected phenotype, but these enzymes are important for resistance to oxidative damage and associated with virulence; thus, they may be of interest for further study among other labs. Interestingly, we identified another locus in *C. lusitaniae* with homology to *ClCAT1* (*CLUG_04072*), *CLUG_05766*; therefore, future studies will be necessary to determine its contribution to oxidative stress resistance in this species. While we chose to knock out catalase-encoding genes to demonstrate the power of the expression-free CRISPR-Cas9 system, the possibilities are endless as to what modification could be made in a diverse array of fungi with this system with minimal tool development.

Many other studies have documented the need for both Cas9 and guide RNA for CRISPR-mediated genome editing. Further, we show that protein, RNA, and DNA can be transformed into cells of *Candida* species via electroporation without prior formation of protoplasts. While we imagine that the protein and RNA are in complex at the time of transformation, this has not been demonstrated in these studies. The ability to transform protein and RNA into *Candida* spp. opens up the possibility of transforming other proteins such as biosensors, enzymes, or inhibitory proteins (31).

## MATERIALS AND METHODS

### Strains and culture conditions.

Strains used in this study, listed in Table S1 in the supplemental material, were grown at 30°C in YPD (2% Bacto peptone, 2% dextrose, 1% yeast extract) with shaking. Transformants were selected on YPD with 200 µg/ml nourseothricin. Cultures were streaked from frozen stocks stored at −80°C in 15% glycerol on a weekly basis.

10.1128/mSphere.00218-17.2 TABLE S1  Strains used in these studies. Download TABLE S1, PDF file, 0.2 MB. Copyright © 2017 Grahl et al. 2017 Grahl et al. This content is distributed under the terms of the Creative Commons Attribution 4.0 International license.

### Plasmids and DNA.

Gene replacement constructs for transformation were generated by fusion PCR. Briefly, 0.5 to 1.0 kb of the 5′ and 3′ regions flanking the gene of interest was amplified from genomic DNA (isolated using the MasterPure yeast DNA purification kit [Epicentre]). The nourseothricin resistance (*NAT1*) cassette was amplified from plasmid pNAT (8). PCR primers used to amplify the three fragments (two flanking regions and *NAT1* gene) contained overlapping homologous sequences as necessary for the subsequent fusion PCR using nested primers (Fig. 1A). The fusion PCR resulted in the *NAT1* cassette flanked by the 5′ and 3′ regions of the gene of interest. PCR products for transformations were purified and concentrated with the Zymo DNA Clean & Concentrator kit (Zymo Research) with a final elution of the construct in molecular-biology-grade water (Corning). One microgram of DNA repair construct was used in each transformation (not including negative controls). All oligonucleotides used in this study can be found in Table S2; primer type corresponds with primer numbers indicated in Fig. 1 to 3.

10.1128/mSphere.00218-17.3 TABLE S2  Sequences of primers and crRNAs used in this study. The sequences in bold represent sequences present in the *NAT1* cassette. Download TABLE S2, PDF file, 0.1 MB. Copyright © 2017 Grahl et al. 2017 Grahl et al. This content is distributed under the terms of the Creative Commons Attribution 4.0 International license.

### Transformation with RNPs.

All transformations were performed by electroporation of competent cells prepared using lithium acetate (LiAc) (32). Overnight cultures with an optical density value at 600 nm (OD_600_) of approximately 1.6 to 2.2 were pelleted and resuspended in 10 ml of transformation buffer (100 mM LiAc, 10 mM Tris-HCl, 1 mM EDTA). These were incubated with shaking for 1 h, before addition of 100 mM dithiothreitol (DTT) for an additional 30 min. After incubation, cells were washed twice with ice-cold water and once with ice-cold 1 M sorbitol before resuspension in approximately 200 μl of ice-cold 1 M sorbitol. Forty microliters of this cell slurry was used per transformation.

RNPs were created using the Alt-R CRISPR-Cas9 system from IDT (Integrated DNA Technologies, Inc.) and assembled for usage during the final washing steps to generate competent cells. Stocks of crRNA (gene specific) and tracrRNA (universal) were dissolved in RNase-free distilled water (dH_2_O; 100 μM) and stored at −20°C. To generate the complete guide RNA, equimolar concentrations (4 μM final) of the gene-specific crRNA and tracrRNA were mixed in water, with a final volume of 3.6 μl per transformation required, and incubated at 95°C for 5 min. Alt-R S.p. Cas9 nuclease 3NLS (60 μM stock from IDT) was diluted to 4 μM in RNase-free dH_2_O, with a final volume of 3 µl per transformation. After the guide RNA (crRNA plus tracrRNA) was allowed to cool to room temperature, it was mixed with diluted Cas9 protein (4 μM) in a 1.2-to-1 ratio (3.6 µl of guide RNA to 3 μl of Cas9 protein) and incubated at room temperature for at least 5 min to assemble the RNP complex. RNPs were used at 6.6 μl per transformation.

Electroporation was performed using an 0.2-cm electroporation cuvette and electroporated with a manual 1.8 pulse (Bio-Rad MicroPulser). Immediately following transformation, cells were resuspended in 1 ml ice-cold 1 M sorbitol and then gently pelleted (3 min, 3,000 rpm) before resuspension in 1 ml liquid YPD. Cells were recovered for 2 to 4 h at 30°C while gently shaking. After recovery, cells were pelleted and resuspended in 200 μl liquid YPD before aliquots were spread plated onto YPD plates with 200 µg/ml nourseothricin and incubated at 30°C for 2 days. To verify nourseothricin resistance, transformants were patched onto selective plates an additional time followed by genotypic and phenotypic characterization.

Negative-control transformation mixtures contained 40 μl of cell slurry and 10 μl of ice-cold 1 M sorbitol (no RNPs, no DNA repair construct). Negative controls did not yield any colonies when plated on YPD plus nourseothricin but yielded robust growth on YPD alone. Minus-RNP (−RNP) transformation mixtures contained 40 μl of cell slurry, 1 μg of DNA repair construct, and 10 μl of ice-cold 1 M sorbitol (no RNPs). Plus-RNP (+RNP) transformation mixtures contained 40 μl of cell slurry, 1 μg of DNA repair construct, 6.6 μl of RNPs, and 10 μl of ice-cold 1 M sorbitol.

The number of NAT^r^ colonies reported in Table 1 represents an average from 3 or 4 independent transformations. Statistics representing the relationship between −RNP and +RNP NAT^r^ colonies were generated using a ratio-paired *t* test.

### Phenotypic assessment.

To assess H_2_O_2_ susceptibility of various *Candida* species with or without deletions of catalase-encoding genes, strains were grown in YPD medium overnight, with aeration by rotating, at 30°C. These cultures were then diluted with fresh medium to an OD_600_ of 1. Serial dilutions of 10-fold were carried out in a microtiter plate to yield seven concentrations ranging from approximately 10^7^ cells/ml (for an OD_600_ of 1) to approximately 10^1^ cells/ml. Ten microliters of each dilution for parental *Candida* strains and catalase null derivatives was applied to YPD plates containing 0 or 3 mM H_2_O_2_. Images were captured after incubation at 37°C for 24 h.
