# Supplementary material for: Use of RNA-Protein Complexes for Genome Editing in Non-albicans Candida Species
Source: mSphere. 2017 Jun 21;2(3):e00218-17. doi: 10.1128/mSphere.00218-17 (PMC5480035; doi:10.1128/mSphere.00218-17)
Supplement: FIG S1 [file sph003172302sf3.pdf]

```
Ca_CAT1                   ------------MAPTFTNSNGQPIPEPFATQRVGQHGPLLLQDFNLIDSLAHFDRERIP
Cg_CTA1                   MSANPTNTSDVRGDRVVTNSTGYPYNDPFATQRVGQHGPLLMQDANLIDSLAHFNRERIP
Cl_CLUG_04072             ------------MAPTFTNSNGCPIPEPFATQRVGKHGPLLLQDFNLIDSLAHFDRERIP
Cau_QG37_05843_05842      -----------MAPPTYTNSQGCPIPEPFATQRVGQHGPLLLQDFNLIDSLAHFDRERIP
                                         . *** * *  :********:*****:** *********:*****
                                      QG37_05843

Ca_CAT1                   ERVVHAKGSGAYGVFEVTDDITDICAAKFLDTVGKKTRIFTRFSTVGGELGSADTARDPR
Cg_CTA1                   ERNPHAHGSGAFGYFEVTDDITDICGSAMFSEIGKRTRFLTRFSTVGGEKGSADTARDPR
Cl_CLUG_04072             ERVVHAKGSGAYGVFEVTDDITDICSSKFLDTVGKKTKVFTRFSTVGGESGSPDTARDPR
Cau_QG37_05843_05842      ERVVHAKGSGAYGVFEVTDDITDICSSKMFEHVGKKTRTFTRFSTVGGESGSADTARDPR
                          **  **:****:* ***********.: ::. :**:*: :********* ** *******

Ca_CAT1                   GFATKFYTEEGNLDLVYNNTPVFFIRDPSKFPHFIHTQKRNPETHLKDANMFWDYLTSNE
Cg_CTA1                   GFATKFYTEEGNLDWVYNNTPIFFIRDPSKFPHFIHTQKRNPQTNLKDPNMFWDYLTTPE
Cl_CLUG_04072             GFSTKLYTEEGNLDLVYNNTPVFFIRDPSKFPHFIHTQKRNPQTHLKDANMFWDYLTSNE
Cau_QG37_05843_05842      GFSTKFYTEEGNLDWVYNNTPVFFIRDPSKFPHFIHTQKRNPETNLKDANMFWDYLCNNP
                          **:**:******** ******:********************:*:*** ******* .

Ca_CAT1                   --ESIHQVMVLFSDRGTPASYREMNGYSGHTYKWSNKKGEWFYVQVHFISDQGIKTLTNE
Cg_CTA1                   NQVAVHQLMILFSDRGTPASYRHMNGYSGHTYKWSNKKGEWHYVQVHILTDQGIKNLTND
Cl_CLUG_04072             --ESVHQVTTLFSDRGTPRSYREMNGYSGHTYKWSTKDGNWHYVQVHFISDQGVKTLTNE
Cau_QG37_05843_05842      --ESIHQVVILFSDRGTPASYREMNGYSGHTYKWSTKDGNWHYVQVHMISDQGNKGLTNE
                             ::**:  ******** ***.************.*.*:*.*****:::*** * ***:
                                                                             QG37_05842

Ca_CAT1                   EAGALAGSNPDYAQEDLFKNIAAGNYPSWTAYIQTMTEAEAKEAEFSVFDLTKVWPHKKY
Cg_CTA1                   EAVEIAGKNPDYCQQDLYENIAKGNYPSWTVYIQTMTEEQAKKLPFSVFDLTKVWPHKDF
Cl_CLUG_04072             EAGELAGSNPDHAQEDLFRNIAKGNFPSWTCYIQTMTAQQAKEAPFSVFDLTKVWPHKDY
Cau_QG37_05843_05842      EATELAGTNPDHAQEDLFKNIAKGNFPSWTCYIQTMTPEQAKQAPFSVFDLTKVWPHKDY
                          **  :**.***:.*:**:.*** **:**** ******  :**:  *************.:

Ca_CAT1                   PLRRFGKFTLNENPKNYFAEVEQAAFSPAHTVPYMEPSADPVLQSRLFSYADTHRHRLGT
Cg_CTA1                   PLRRVGKMVMNENPLNYFASIEQAAFSPANTVPYQEPSADPVLQSRLFAYADAHRYRLGA
Cl_CLUG_04072             PLRRFGKLTLNENPKNYFAEVEQAAFSPAHTVPYMEPSADPVLQSRLFSYADTHRHRLGP
Cau_QG37_05843_05842      PMRRFGKLTLNENPKNYFAEVEQAAFSPSHTVPYQEASADPVLQSRLFSYPDTHRHRLGT
                          *:**.**:.:**** ****.:*******::**** * ***********:* *:**:***

Ca_CAT1                   NYTQIPVNCPVTGAVFNPHMRDGAMTVNGNLGSHPNYLASDKPVEF--KQFSLQEDQEVW
Cg_CTA1                   NFHQIPVNCPYASKFFNPVIRDGPMNVDGNFGNEPNYYASNKQYQIPNQDRPIQQHQEVW
Cl_CLUG_04072             NYTQIPVNCPITG-VYNPHMRDGAMNVNGNLGSAPNYLATSEPIEF--RQFSIQEDQEVW
Cau_QG37_05843_05842      NYSQIPVNCPVASQVYAPHIRNGAMTVNGNLGSTPNYVSSFNPMQF--REFSQQQDQEVW
                          *: ******* :. .: * :*:* *.*:**:*. *** :: :  ::  ::   *:.****

Ca_CAT1                   NGAATPFHWKATPA--DFKQAQELWKVLKRYPNQQEHLAHNIAVHAAGADAAIQDRVFAY
Cg_CTA1                   NGPAQPFHWATSPGDIDFVQARDLYKVLGKQKDQQEHLAYNIGTHVAGADPEIQQRVIDM
Cl_CLUG_04072             NGAACPFHWKATED--EFTQATALYNVLGRYPGQQKNLAHNVAVHVSAADKPIQERVIKY
Cau_QG37_05843_05842      TGAACPFHWKATDA--EYTQATALYHVLAKYPNQQRNLAHNVAVHAAGADPSVQDRVFEL
                          .* * **** ::    :: **  *::** :  .**.:**:*:..*.:.**  :*:**:

Ca_CAT1                   FGKVSQDLADAIKKEVLELSPRK*----
Cg_CTA1                   FSRVDEGLGANIRKEIEKNASAPTAKI*
Cl_CLUG_04072             FGKVSPELAANIRKELEAN*--------
Cau_QG37_05843_05842      FTRVHPDLGAAIKKEVLELSPRK*----
                          * :*   *.  *:**:
```

**Figure S1.Alignment to compare the catalase protein sequences of *C. lusitaniae*, *C. glabrata* and *C. auris* to *Candida albicans* Cat1.** Protein sequences were retrieved from the *Candida* genome database or NCBI and sequences were aligned using the Clustal Omega multiple sequence alignment tool. The two *C. auris* ORF annotations that made up the *CAT1* gene are indicated in red (*QG37_05843*) or blue (*QG37_05842*).
