# Supplementary material for: Use of RNA-Protein Complexes for Genome Editing in Non-albicans Candida Species
Source: mSphere. 2017 Jun 21;2(3):e00218-17. doi: 10.1128/mSphere.00218-17 (PMC5480035; doi:10.1128/mSphere.00218-17)
Supplement: TABLE S1 [file sph003172302st1.pdf]

Table S1. Strains used in these studies.

Strain	Lab #	Genotype	Source
C. lusitaniae A04 (haploid)	DH2949	Parent, Nat^S	Dartmouth Hitchcock Medical Center
C. auris CAU-10	DH2777	Parent, Nat^S	Centers for Disease Control
C. glabrata ATCC2001	DH2788	Parent, Nat^S	Scott Moye-Rowley
*C. lusitaniae cat1*Δ-a	DH2945	Deletion of *CLUG_04072*, Nat^R, clone 6	This study
*C. lusitaniae cat1*Δ-b	DH2946	Deletion of *CLUG_04072*, Nat^R, clone 13	This study
*C. lusitaniae cat1*Δ-c	DH2947	Deletion of *CLUG_04072*, Nat^R, clone 18	This study
*C. lusitaniae cat1*Δ-d	DH2948	Deletion of *CLUG_04072*, Nat^R, clone 21	This study
*C. auris cat1*Δ-a	DH2950	Deletion of QG_05843-05842, Nat^R, clone 1	This study
*C. auris cat1*Δ-b	DH2951	Deletion of QG_05843-05842, Nat^R, clone 3	This study
*C. auris cat1*Δ-c	DH2952	Deletion of QG_05843-05842, Nat^R, clone 5	This study
*C. auris cat1*Δ-d	DH2953	Deletion of QG_05843-05842, Nat^R, clone 10	This study
*C. glabrata cta1*Δ-a	DH2954	Deletion of *CTA1*, Nat^R, clone 4	This study
*C. glabrata cta1*Δ-b	DH2955	Deletion of *CTA1*, Nat^R, clone 5	This study
*C. glabrata cta1*Δ-c	DH2956	Deletion of *CTA1*, Nat^R, clone 7	This study
*C. glabrata cta1*Δ-d	DH2957	Deletion of *CTA1*, Nat^R, clone 8	This study
