# Supplementary material for: Use of RNA-Protein Complexes for Genome Editing in Non-albicans Candida Species
Source: mSphere. 2017 Jun 21;2(3):e00218-17. doi: 10.1128/mSphere.00218-17 (PMC5480035; doi:10.1128/mSphere.00218-17)
Supplement: TABLE S2 [file sph003172302st2.pdf]

Table S2. Sequences of primers and crRNAs used in this study. The sequences in bold represent sequences present in the *NAT1* cassette.

Primer Type	name	gene	sequence
9	NG_087	*NAT1*	GAAGTTCCAGTTGATCCACCATTGA
10	rev seq NAT1	*NAT1*	CGATGGTACTGCTTCCGATGG
1	NG_128_CLUG_04072_left flank_fw	*Candida lusitaniae CLUG_04072*	AGAGGTAGTTACTAACAAGGAAG
2	NG_129_CLUG_04072_left flank rev	*Candida lusitaniae CLUG_04072*	**AACGTCGTGACTGGGAAAaaTCATTA**CTTACTCATGCTGACGCAACTA
5	NG_130_CLUG_04072_CRISPR fw	*Candida lusitaniae CLUG_04072*	TAGTTGCGTCAGCATGAGTAAG**TAATGAttTTTCCCAGTCACGACGTT**
6	NG_131_CLUG_04072_CRISPR rev	*Candida lusitaniae CLUG_04072*	TCCTTTGCTGGTCTCGTATTTC**GTGGAATTGTGAGCGGATA**
3	NG_132_CLUG_04072_right flank fw	*Candida lusitaniae CLUG_04072*	**TATCCGCTCACAATTCCAC**GAAATACGAGACCAGCAAAGGA
4	NG_133_CLUG_04072_right flank rev	*Candida lusitaniae CLUG_04072*	AGGCTTACACGTTAGACTGTTC
7	NG_134_CLUG_04072_CRISPR_nested_fw	*Candida lusitaniae CLUG_04072*	AAGTTACCGTGTTTGCATTTCTC
8	NG_135_CLUG_04072_CRISRP_nested_rev	*Candida lusitaniae CLUG_04072*	CTGCTCTCTAAGGTCATAGCATAAA
1	NG_140_Cg_CTA1_left flank_fw	*Candida glabrata CTA1*	CTGGTCGTTCAACTGAGAAAGT
2	NG_141_Cg_CTA1_left flank rev	*Candida glabrata CTA1*	**AACGTCGTGACTGGGAAAaaTCATTA**AGGATAAAGGATGTGTTCCAAGG
5	NG_142_Cg_CTA1_CRISPR fw	*Candida glabrata CTA1*	CCTTGGAACACATCCTTTATCCT**TAATGAttTTTCCCAGTCACGACGTT**
6	NG_143_Cg_CTA1_CRISPR rev	*Candida glabrata CTA1*	TTTACAGCACTTTACGTGGTTC**GTGGAATTGTGAGCGGATA**
3	NG_144_Cg_CTA1_right flank fw	*Candida glabrata CTA1*	**TATCCGCTCACAATTCCAC**GAACCACGTAAAGTGCTGTAAA
4	NG_145_Cg_CTA1_right flank rev	*Candida glabrata CTA1*	CCTGCGTTGTTAACTGTCTTG
7	NG_146_Cg_CTA1_CRISPR_nested_fw	*Candida glabrata CTA1*	AGAAAGTTCCAGCTTCTAAGCTATT
8	NG_147_Cg_CTA1_CRISRP_nested_rev	*Candida glabrata CTA1*	AGGCTCTTGTATGGTTTGCTC
1	NG_150_Cau_left flank_fw	*Candida auris Q37_05842_05843*	CTGAGCAGCAACTACTCATACA
2	NG_151_Cau_left flank rev	*Candida auris Q37_05842_05843*	**AACGTCGTGACTGGGAAAaaTCATTA**GGCAGCCTTTATATACCCATGA
5	NG_152_Cau_05842_05843_CRISPR fw	*Candida auris Q37_05842_05843*	TCATGGGTATATAAAGGCTGCC**TAATGAttTTTCCCAGTCACGACGTT**
6	NG_153_Cau_05842_05843_CRISPR rev	*Candida auris Q37_05842_05843*	TTACATCACGTAGGCTTGTAGC**GTGGAATTGTGAGCGGATA**
3	NG_154_Cau_right flank fw	*Candida auris Q37_05842_05843*	**TATCCGCTCACAATTCCAC**GCTACAAGCCTACGTGATGTAA
4	NG_155_Cau_right flank rev	*Candida auris Q37_05842_05843*	GCTACGTTGCTGGTACTACTAAG
7	NG_156_Cau_CRISPR_nested_fw	*Candida auris Q37_05842_05843*	GCAGAAATCTGACCTGACACT
8	NG_157_Cau_CRISRP_nested_rev	*Candida auris Q37_05842_05843*	ATCACCTTCTTTCCTTCCTCATC
**cRNA**	**name**	**gene**	**sequence**
	crRNA_CLUG_04072	*Candida lusitaniae CLUG_04072*	TTTGCGTGTGGATAAAGTGG
	crRNA_Cg_CTA1	*Candida glabrata CTA1*	ACCAGATTCTCCACTGTCGG
	crRNA_Cau_05842_3	*Candida auris Q37_05842_05843*	ACTAGATTCTCCACCGTCGG
